# Roles and Impacts of Integrative Medical Interventions in Central Nervous System Tumor Treatment: Multi‐Technology Convergence and the Paradigm Shift Toward Functional Reconstruction

**DOI:** 10.1111/cns.70516

**Published:** 2025-07-14

**Authors:** Junda Lai, Ketao Liu, Yuhua Lin, Zhonglin Chen, Xianglun Ji, Junkai Wen

**Affiliations:** ^1^ Fujian University of Traditional Chinese Medicine Fujian China; ^2^ Xiamen TCM Hospital Affiliated to Fujian University of Traditional Chinese Medicine Xiamen China; ^3^ Shanghai University of Traditional Chinese Medicine Shanghai China

**Keywords:** artificial intelligence, central nervous system tumor, functional reconstruction, integrative medicine, multi‐technology convergence

## Abstract

**Aims:**

Central nervous system (CNS) tumors, particularly glioblastoma, remain a formidable clinical challenge due to their aggressive nature, high heterogeneity, and the restrictive blood–brain barrier. This review aimed to synthesize the roles and impacts of integrative medical interventions in CNS tumor management, highlighting the evolution from traditional monotherapy to multi‐technology convergence. It further seeks to address technical barriers and ethical considerations to guide future research and clinical innovation in this field.

**Methods:**

The review discusses a range of integrative medical interventions, including noninvasive neuromodulation, electrotherapy, phototherapy, ultrasound, and magnetotherapy, alongside psychological, nutritional, and herbal medicine approaches. It also evaluates advanced technologies reshaping precision oncology, such as AI‐driven diagnostics, 3D organoid models, and multi‐omics sequencing, while examining barriers to technical integration and ethical governance issues.

**Results:**

Integrative medical interventions, combined with multi‐technology convergence, play significant roles in CNS tumor management: noninvasive neuromodulation, electrotherapy, phototherapy, ultrasound, and magnetotherapy, together with psychological, nutritional, and herbal approaches, contribute to comprehensive care. Advanced technologies—including AI‐driven diagnostics, 3D organoid models, and multi‐omics sequencing—enable dynamic disease monitoring, targeted drug delivery, and immune microenvironment remodeling, thereby advancing precision oncology. However, technical integration is hindered by data heterogeneity and translational inefficiency, necessitating standardized frameworks and interdisciplinary collaboration. Additionally, ethical governance is critical for ensuring equitable care.

**Conclusion:**

The future landscape of integrative CNS tumor management lies in achieving functional reconstruction through technology‐driven innovation, patient‐centered care, and global cooperation, with standardized frameworks and interdisciplinary collaboration essential to overcoming existing challenges.

## Graphical abstract

Multi‐technology interventions (non‐invasive neuromodulation, AI, 3D organoids) for CNS tumor treatment shift from monotherapy to functional reconstruction. They address BBB restriction, tumor heterogeneity, and immune suppression via biological–psychological–technological convergence, enabling precision oncology through dynamic monitoring and microenvironment remodeling. Despite translational/ethical challenges, interdisciplinary innovation drives patient‐centered functional restoration.

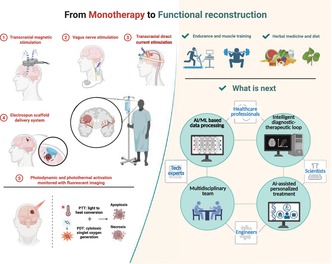

**Abbreviations**
ACT: acceptance and commitment therapy
AMF: alternating magnetic fields
AR: Astragalus radix
BBB: blood–brain barrier
BDNF: brain‐derived neurotrophic factor
Bn: Bombesin
BnR: Bn‐related peptides and their receptors
BsAbs: bispecific antibodies
BTB: blood–tumor barrier
BTOs: brain tumor organoids
CBT: cognitive behavioral therapy
CIH: Comprehensive Integrated Health
CN: central neurocytoma
CNS: central nervous system
DMG: diffuse midline glioma
DTI: diffusion tensor imaging
ECM: extracellular matrix
EO: essential oil
EV: extracellular vesicle
FGR: fluorescence‐guided resection
FUS: focused ultrasound
GAM: glioma‐associated microglia/macrophage
GBM: glioblastoma
GLAD: glioblastoma ketogenic adjuvant diet
GSC: glioblastoma stem cells
HGG: high‐grade glioma
ICG: indocyanine green
KD: ketogenic diet
KR: KillerRed
LGG: low‐grade glioma
MDT: multidisciplinary team
MHT: magnetic hyperthermia therapy
MNPs: magnetic nanoparticles
MPI: magnetic particle imaging
MTR: magnetization transfer ratio
MΦ: macrophage
nab‐PTX: Nab‐Paclitaxel
NGS: next‐generation sequencing
NIR: near‐infrared
nTMS: navigation transcranial magnetic stimulation
OS: overall survival
Ov: oncolytic virus
PACTS: physical activity in childhood tumor survivors
PBT: primary brain tumor
PCNSL: primary central nervous system lymphoma
PDT: photodynamic therapy
PFBT: posterior fossa brain tumors
PFS: progression‐free survival
PTX: paclitaxel
QoL: quality of life
QST: quinazolin‐4(3H)‐one
RCTs: randomized controlled trials
RTK: receptor tyrosine kinase
RWD: real‐world data
SAG: sagittal
SAHA: suberoylanilide hydroxamic acid
SERS: surface‐enhanced Raman scattering
SMA: supplementary motor area
SN:
*Solanum nigrum*

SRS: stereotactic radiosurgery
tACS: transcranial alternating current stimulation
TAM: tumor‐associated macrophage
tDCS: transcranial direct current stimulation
TME: tumor microenvironment
TMS: transcranial magnetic stimulation
TMZ: temozolomide
TRA: transverse
TTFields: tumor treating fields
VNS: vagus nerve stimulation
WES: whole exome sequencing
WGS: whole genome sequencing
WM: white matter
YB: Yunnan Baiyao
ZM: PTPRZ1‐MET

## 1 Introduction

Glioblastoma (GBM), a grade IV glioma of the central nervous system (CNS), represents one of the most aggressive malignancies in clinical oncology. With an incidence rate of 3.19 cases per 100,000 individuals [1], GBM accounts for 49.1% of all malignant brain tumors, yet yields a dismal 5‐year survival rate of only 6.8% in the United States [2]. Even with the current standard treatment, surgical resection followed by radiotherapy and temozolomide (TMZ) chemotherapy, the median survival of affected patients remains < 15 months, underscoring the near incurability of this disease due to its inherent propensity for recurrence and fatal progression. This recalcitrance is rooted in the tumor's profound biological complexity, marked by intertumoral heterogeneity and an immunosuppressive [3].

Current therapeutic modalities, including surgical resection, radiotherapy, and TMZ‐based cytotoxic therapy, achieve only marginal survival benefits. While surgery remains critical for local tumor control, its clinical utility is constrained by anatomical complexity and postoperative morbidity. For example, radical resection of complex meningiomas combined with stereotactic radiosurgery (SRS) achieves > 90% tumor control rates but results in variable neurological outcomes (8%–66% improvement) and complication rates ranging from 3%–40% [4]. In SRS, a tumor margin dose of 12–15 Gy reduces complication rates to 0%–16%, yet the safe threshold for optic nerve irradiation remains undefined. Low‐dose irradiation protocols may still induce vision loss, with a strong correlation observed between the length of Cranial Nerve (CN) II irradiation and postoperative visual decline. For intraparenchymal tumors involving the supplementary motor area (SMA), postoperative SMA syndrome occurs in up to 70% of cases, manifesting as acute motor dysfunction. Parasagittal meningioma resection carries additional risks of cerebrospinal fluid leakage and venous infarction, particularly in patients with preoperative cognitive or motor deficits [5, 6]. Radiotherapy and chemotherapy also face significant limitations. Since the introduction of the Stupp protocol in 2005, TMZ has extended median survival to 14 months in MGMT promoter‐methylated patients, yet adaptive resistance driven by tumor heterogeneity persists [1]. Further improvements in GBM treatment outcomes are hindered by multiple barriers, including the tumor's infiltrative nature, immunosuppressive “cold” microenvironment, and the restrictive role of the blood–brain barrier (BBB) [7]. Additionally, postoperative cognitive dysfunction (a well‐documented CNS complication of anesthesia and surgery) impairs patients' social and cognitive abilities, leading to personality changes, memory deficits, and other sequelae [8].

Collectively, the high lethality, therapeutic complexity, and limitations of conventional approaches underscore the urgent need for novel treatment strategies. Integrative medical interventions incorporating cutting‐edge technologies may represent a critical future direction for CNS tumor management. Advances in integrative medical interventions, particularly those incorporating cutting‐edge technologies, offer promising avenues for overcoming the multifaceted barriers in CNS tumor treatment. This review synthesizes current evidence on non‐invasive neuromodulation, electrotherapy, phototherapy, and complementary psychological/nutritional approaches, while also evaluating emerging technologies such as AI‐driven diagnostics, 3D organoid modeling, and multi‐omics platforms. By addressing technical challenges (e.g., data heterogeneity, translational inefficiency) and ethical considerations (e.g., data privacy, AI transparency), this analysis aims to guide future research and clinical innovation in CNS tumors.

## 2 Integrative Medicine: A Multidimensional Medical Model Integrating Biological, Psychological, Social, and Technological Interventions

The evolution of medical philosophy is reshaping clinical practice paradigms. When confronting complex symptom clusters, singular diagnostic models often prove inadequate. Research in functional and psychosomatic medicine has confirmed the existence of multifactorial interactions in most disease processes. Physicians increasingly recognize that their therapeutic focus extends beyond the initial pathological lesion to encompass the entire trajectory of disease progression. While pharmacological interventions targeting specific lesions may alleviate local symptoms, they fail to address patients' holistic health needs. This cognitive shift underscores the necessity of integrating biological–psychological–social factors in integrative medicine, which enhances therapeutic efficacy and mitigates potential side effects of fragmented interventions [9].

In high‐income countries, 5‐year survival rates for brain tumor patients vary significantly according to histopathological type. For example, patients with meningioma achieve an 88% 5‐year survival rate, compared to only 7% for GBM patients. Regardless of treatment modality, brain tumor survivors frequently encounter multidimensional challenges (physical, psychological, and cognitive), which call for survivorship care that supports daily functioning, emotional well‐being, and social participation [10].

Against this backdrop, the clinical value of integrative medicine has gained prominence. Systemic self‐management interventions provide comprehensive support for symptom control, lifestyle modification, and psychosocial needs. Evidence‐based dietary guidance, exercise prescriptions, and emotional regulation protocols have demonstrated efficacy in improving quality of life. Concurrently, digital health solutions (leveraging electronic information systems, smart devices, and remote monitoring technologies) can overcome geographical, physical, and social barriers inherent in traditional face‐to‐face care. These supports can be provided through self‐guided platforms or with professional facilitation, offering efficient solutions for patients' daily needs.

**FIGURE 1:**
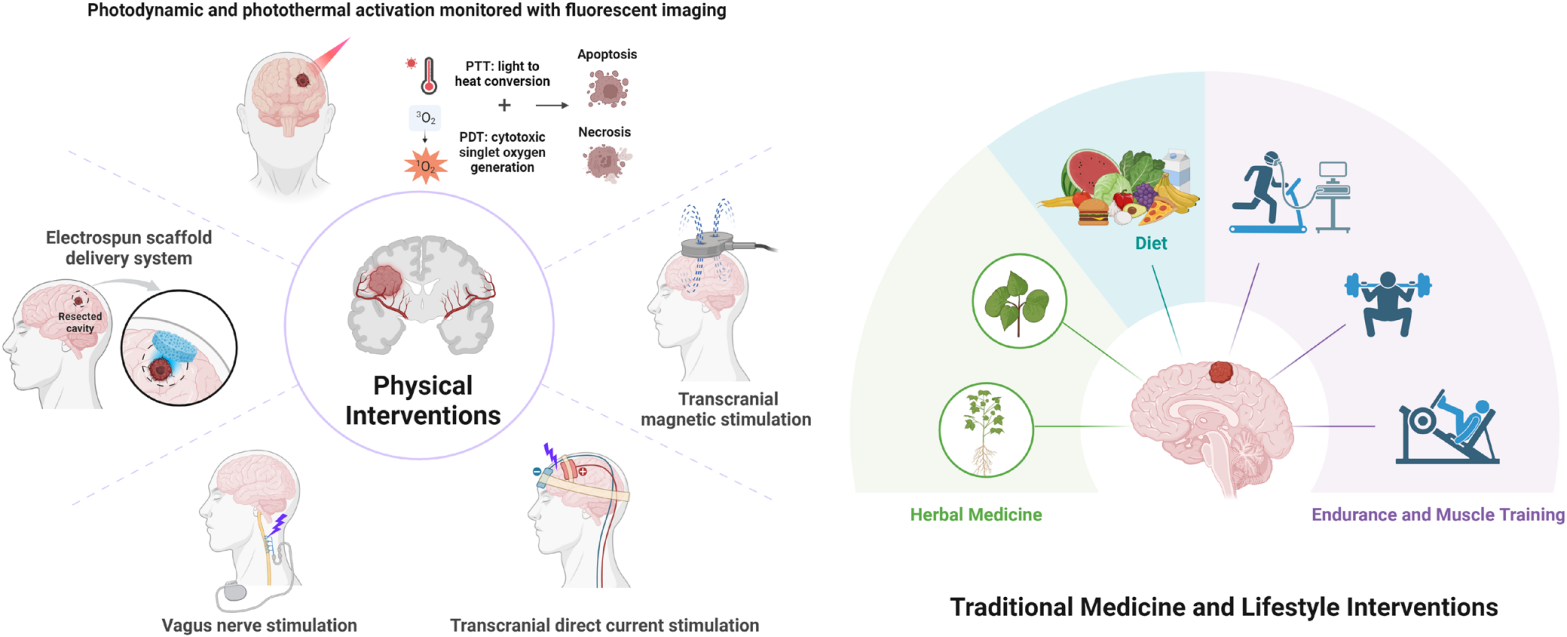
Integrative medicine framework for CNS tumor management. The integrative medical model for CNS tumor care combines biological, psychological, social, and technological interventions to address multidimensional challenges. This framework emphasizes patient‐centered functional reconstruction through synergistic collaboration across domains.

In summary, CNS tumor management is undergoing a paradigmatic shift from disease‐centric intervention to life‐course health maintenance. Integrative medicine, as a multidimensional model integrating biological, psychological, social, and technological interventions, provides an innovative pathway to improve patient outcomes. Its core framework encompasses non‐pharmacological therapies, lifestyle modification, and technological synergy, aiming to achieve comprehensive enhancement from disease control to functional restoration through the establishment of a patient‐centered Comprehensive Integrated Health (CIH) ecosystem (Figure 1).

Notably, the patient‐centered CIH model, which exemplifies the principles of integrative medicine, has shown significant value in the multidisciplinary rehabilitation of chronic pain and complex conditions. Well‐established systems within large healthcare networks, such as the U.S. Department of Veterans Affairs, have not only improved patient satisfaction but also optimized opioid reduction strategies and enhanced healthcare cost‐effectiveness. Crucially, the high feasibility of implementing CIH programs via telemedicine has expanded treatment access for multimorbid patients, further validating the potential of integrative medicine to enhance therapeutic efficacy, reduce complications, and improve the quality of life [11].

## 3 Traditional Core Modules of Integrative Medical Interventions

### 3.1 Non‐Invasive Neuromodulation Techniques

Non‐invasive brain stimulation technologies, characterized by their ability to precisely modulate neural activity and plasticity, play a dual role in advancing CNS tumor research and therapy. These techniques not only decode the molecular mechanisms underlying tumor–neuron electrical signal interactions but also exhibit multidimensional therapeutic potential by reshaping the tumor microenvironment (TME). Through targeted regulation of stromal cells, immune cells, and the extracellular matrix within the TME, they offer a novel paradigm for precision management of CNS tumors [12]. This technical system encompasses transcranial magnetic stimulation (TMS), transcranial direct current stimulation (tDCS), transcranial alternating current stimulation (tACS), and vagus nerve stimulation (VNS), enabling dynamic remodeling of neural functional networks without breaching the BBB. In postoperative neurological rehabilitation, interventional neurorehabilitation using non‐invasive neuromodulation (e.g., TMS, tDCS, and continuous cortical electrical stimulation) has emerged as a critical strategy to improve functional deficits in glioma patients.

A multicenter analysis integrating data from 2 randomized controlled trials (RCTs) and 3 single‐arm studies demonstrated that low‐frequency contralesional navigated repetitive TMS, combined with physiotherapy, significantly improved motor function and functional status. Linear mixed models revealed that the treatment group exhibited superior British Medical Research Council scores (7 days: OR 3.28, 95% CI: 1.08–9.99; 3 months: OR 2.03, 95% CI: 0.65–6.39) and Karnofsky Performance Scale scores (7 days: MD 11, 95% CI: 2–19; 3 months: MD 11, 95% CI: 0‐2) compared to the sham group. Subgroup analysis showed a stronger treatment effect in patients with postoperative MRI‐proven ischemia, and seven clinical studies (*n* = 118) further demonstrated that non‐invasive stimulation significantly ameliorates motor and language impairments, with fMRI evidence of functional reorganization in the motor cortex observed in select cases [13, 14]. Notably, Shi and Wu [15] identified shared analgesic mechanisms: modulation of ion channels, neurotransmitters, and inflammatory responses to inhibit pain conduction, while activating endogenous analgesic pathways and promoting neuroplasticity to prevent central sensitization. Despite supportive evidence, long‐term effects on cognitive network regulation require validation through multicenter RCTs.

For preoperative functional mapping, navigated transcranial magnetic stimulation (nTMS) integrates MRI with three‐dimensional spatial localization technology to achieve millimeter‐level precision in motor cortex mapping. Riaz et al. [16] demonstrated that nTMS combined with MRI optimizes surgical trajectory design and predicts postoperative neurological deficit risks. Muir et al. [17] combined nTMS‐diffusion tensor imaging (DTI) evaluation model revealed that 46.7% of motor‐area glioma patients exhibit nTMS hotspot shifts, with predictive efficacy significantly superior to traditional anatomical grading criteria. However, Eibl et al.'s [18] study on meningiomas showed limited predictive value of nTMS for postoperative motor outcomes (no statistically significant difference in motor dysfunction rates between groups), underscoring the necessity to establish multi‐modal preoperative assessment systems integrating intraoperative electrophysiological monitoring and postoperative functional imaging follow‐up.

With advancements in precision medicine, non‐invasive neuromodulation is evolving from a standalone therapy to an integrated diagnostic and therapeutic system. Its clinical value extends beyond neuroprotection and reconstruction, providing new dimensions for personalized surgical planning, postoperative rehabilitation design, and optimization of drug delivery systems through real‐time monitoring of neuroplasticity dynamics. Future research should focus on technology standardization, multi‐modal data fusion, and long‐term efficacy evaluation to facilitate the translation of this field from experimental studies to clinical decision support systems [19].

### 3.2 Electrotherapy

The highly invasive nature of malignant CNS tumors poses significant challenges for improving survival outcomes. Recent studies have revealed pathological electrophysiological coupling between high‐grade gliomas and adjacent healthy brain tissues. This aberrant coupling promotes tumor progression by enhancing cancer cell invasion and angiogenesis, thereby providing novel targets for electrical modulation therapy.

Among treatment modalities, tumor‐treating fields (TTFields) have emerged as an innovative therapy for high‐grade CNS tumors lacking effective alternatives. This technology exerts its effects by disrupting cancer cell mitosis through low‐intensity alternating electric fields, thereby inducing apoptosis. The FDA‐approved Optune system has been successfully applied in clinical settings for primary and recurrent GBM, serving as a critical adjunct to surgery, radiotherapy, and chemotherapy.

Branter et al.'s groundbreaking research systematically analyzed the genome‐wide impacts of electrotherapy (TTFields and deep brain stimulation) on brain tumor cells. Results demonstrated that deep brain stimulation inhibits proliferation via G0 cell cycle arrest, while TTFields act through G2 phase blockade. Both modalities exhibit synergistic effects when combined with chemotherapy, with differentially expressed genes significantly enriched in pathways related to mitochondrial dysfunction and endoplasmic reticulum stress. These findings elucidate dual mechanisms by which electrotherapy disrupts tumor cell energy metabolism and protein homeostasis, laying a molecular foundation for combined electrical modulation therapy in adult and pediatric high‐grade brain tumors [20].

In the treatment of high‐grade glioma (HGG), Zhang et al.'s single‐center retrospective study provided the first clinical evidence for TTFields. The addition of TTFields to standard therapy extended median progression‐free survival (PFS) to 14.2 months (vs. 10.3 months in controls) and overall survival (OS) to 19.7 months (vs. 15.5 months in controls), with no significant difference in adverse event rates. After controlling for confounding factors via propensity score matching, results remained robust, confirming the efficacy and safety of TTFields in HGG management [21].

For GBM combination therapy, the synergistic effects of TTFields and TMZ have been clinically validated. Hila Fishman et al.'s mechanistic study revealed that TTFields induce BRCA‐deficient states by downregulating the FA‐BRCA pathway, enhancing sensitivity to DNA damage. This effect is particularly pronounced in MGMT promoter‐unmethylated cells, where TTFields combined with TMZ/CCNU produce synergistic outcomes, whereas additive effects are observed in MGMT‐methylated cells. These findings explain the differential efficacy of TTFields‐based regimens across molecular subtypes of GBM, providing a theoretical basis for personalized treatment [22].

Although the phase III EF‐14 trial confirmed equivalence between TTFields plus TMZ and TMZ monotherapy for maintenance treatment, clinical practices in China remain controversial. Chen et al.'s retrospective analysis of 267 GBM patients demonstrated that the combination therapy group achieved a median OS of 18.9 months, significantly superior to 14.6 months in controls. Multivariate analysis identified treatment adherence (> 70% device wear time) and complete Stupp protocol implementation as key factors associated with improved outcomes. This study not only validated the generalizability of international trial results but also uncovered unique benefit patterns in the Chinese patient population through subgroup analysis, providing evidence‐based support for optimizing clinical applications [23].

### 3.3 Phototherapy

Malignant brain tumors, characterized by high heterogeneity and poor prognosis in the CNS, present significant challenges in clinical management. Recent advancements in near‐infrared (NIR) optical imaging techniques (fluorescence lifetime imaging/photoacoustic imaging) have provided critical technical support for precision oncology, leveraging their non‐invasive and high‐resolution capabilities. While single‐modal imaging enables tumor characterization, multi‐modal integration (dual/triple‐modal) further enhances localization accuracy. At the therapeutic level, NIR‐guided combination therapies, such as photothermal therapy combined with chemotherapy, offer innovative solutions to overcome BBB limitations through synergistic multimechanistic effects [24].

Fluorescence‐guided resection (FGR) and photodynamic therapy (PDT) have emerged as research hotspots. Ren et al.'s systematic review and meta‐analysis of 1294 patients revealed that FGR achieved a gross total resection rate of 73.00%, while PDT‐treated patients demonstrated a median OS of 17.78 months, PFS of 10.82 months, and 1‐ and 2‐year survival rates of 59% and 25%, respectively. These findings indicate that FGR improves resection rates and PDT prolongs survival, though large‐sample randomized controlled trials are needed to validate outcomes due to limited existing evidence [25].

The synergistic application of oncolytic viruses and PDT has opened new avenues for treating malignant CNS tumors. Shimizu et al. developed the oncolytic herpes simplex virus G47Δ‐KR expressing the photosensitizer KillerRed, achieving tumor cytotoxicity through dual mechanisms of viral targeting and light activation. In vitro experiments showed significantly enhanced tumor cell killing efficiency under irradiation compared to non‐irradiated controls. In vivo studies confirmed superior inhibition of GBM and malignant melanoma xenografts compared to monotherapy. Mechanistic investigations revealed that this combination therapy remodels the tumor microenvironment by enhancing immune cell infiltration, providing a novel strategy to overcome CNS tumor immunosuppression [26].

For targeted glioma therapy, Wang et al. designed an extracellular vesicle (EV) system loaded with indocyanine green (ICG) and paclitaxel (PTX) (ICG/PTX@RGE‐EV). Under 808 nm laser irradiation, this system exhibited robust photothermal properties, effectively promoting PTX release and targeting U251 cells. The combination of chemotherapy and thermotherapy induced apoptosis via caspase‐3 pathway activation, prolonging median survival time by 48 days compared to the control group. These results suggest that ICG/PTX@RGE‐EV may inhibit glioma growth through a chemo–thermo combined targeting strategy [27].

Current phototherapy research demonstrates a trend toward multi‐technology synergy and mechanism integration. Future breakthroughs should focus on optimizing treatment protocols, expanding clinical sample sizes, and exploring novel targeted delivery systems to advance malignant brain tumor therapy into an era of precision and personalization.

### 3.4 Ultrasound Therapy

The BBB penetration barrier represents a central challenge in brain tumor treatment. Traditional nanodrug modification strategies are limited by the complex brain microenvironment, whereas ultrasound‐assisted brain delivery technology, in synergy with specific nanodrugs, enables safe and reversible BBB disruption, significantly enhancing drug penetration into the brain. This innovation provides a novel pathway to overcome therapeutic bottlenecks [28].

Focused ultrasound (FUS) technology has demonstrated unique advantages in treating diffuse midline glioma (DMG, previously known as DIPG). As a lethal pediatric brain tumor, DMG has resisted over 250 clinical trials due to the high compactness of the BBB and blood–tumor barrier (BTB). FUS uses ultrasonic energy at specific frequencies to achieve transient BBB permeabilization or even disruption, concomitantly promoting therapeutic drug delivery into the tumor microenvironment. Preclinical studies show that this technology enables effective accumulation of otherwise impermeable drugs in tumor regions, offering new hope for DMG treatment [29].

Current FUS technology has entered the clinical translation phase: preclinical DMG models have demonstrated significant efficacy when combined with chemotherapy, and clinical trials for pediatric brain tumors are ongoing. Notably, FUS also exhibits multiple therapeutic modalities, including sonodynamic therapy and radiosensitization, further expanding its application scenarios in malignant brain tumor treatment [30]. The technology's ability to non‐invasively breach biological barriers provides an innovative solution to tackle brain tumor treatment challenges. Future research should focus on optimizing treatment parameters, exploring multi‐modal combinations, and evaluating long‐term safety.

### 3.5 Magnetotherapy

Primary central nervous system lymphoma (PCNSL), a rare extranodal non‐Hodgkin lymphoma, poses significant challenges in diagnosis and therapeutic evaluation. It predominantly affects the white matter of the brain and spinal cord or deep‐seated brain structures. Traditional contrast‐enhanced MRI is the standard diagnostic modality, but its results can be confounded by other brain pathologies. Although FDG–PET has diagnostic value, atypical lymphoma presentations can make interpretation difficult. Studies have indicated that combined PET–MR systems significantly improve the efficacy of lesion detection, offering a novel tool for precise diagnosis of PCNSL [31].

Radiation necrosis, a rare complication of craniospinal irradiation in pediatric medulloblastoma patients, can be evaluated using magnetization transfer ratio (MTR) measurements of macromolecular content. Harreld et al. analyzed the correlations between supratentorial MTR and radiation necrosis in 95 medulloblastoma patients through pre‐radiotherapy baseline MR imaging. Among the 23 children who developed radiation necrosis after treatment, the pre‐treatment supratentorial white matter (WM) MTR values were significantly lower, regardless of age. These findings suggest that lower baseline supratentorial WM MTR may help identify children at risk of radiation necrosis following radiotherapy [32].

Two emerging therapeutic approaches within magnetotherapy hold promise. Magnetic hyperthermia therapy (MHT) is a novel treatment for brain tumors. It involves the local delivery of magnetic nanoparticles (MNPs) to the brain and activation by an external alternating magnetic field (AMF) to induce hyperthermia. Clinical and preclinical trials suggest that with technological advancements, MHT may enhance the efficacy of radiotherapy and chemotherapy. The key to its clinical success lies in optimized MNP design, reliable local delivery methods, and AMF systems integrated with magnetic particle imaging (MPI) [33]. Thirumurugan et al. synthesized FeTi@Au‐ANG NPs for glioma hyperthermia. These spherical ~16 nm nanoparticles generate heat under AMF stimulation for localized thermal ablation. In vitro experiments demonstrated effective glioma cell targeting, increased apoptosis, and high brain tissue deposition, yielding favorable outcomes in orthotopic intracranial xenograft mouse models. This represents a promising targeted delivery strategy for glioma treatment [34].

### 3.6 Cognitive Behavioral Therapy (CBT) and Acceptance and Commitment Therapy (ACT)

The psychological and physiological well‐being of patients with primary malignant brain tumors and their caregivers is significantly impacted during diagnosis and treatment. Interventions such as CBT and ACT have shown promise in addressing these challenges, particularly in managing symptoms like fatigue, anxiety, and caregiving burden.

Fatigue is a prevalent and debilitating symptom in patients with diffuse glioma, yet evidence‐based treatments for severe fatigue remain limited. Building on CBT's established efficacy in reducing fatigue in other populations, Röttgering et al. conducted a randomized controlled trial involving 100 patients with stable disease and fatigue severity scale scores ≥ 35. Participants were randomized to receive a 12‐week intervention combining in‐person sessions and web‐based modules. The study employed multi‐metric assessments, magnetoencephalography/MRI biomarker exploration, and Bayesian design to evaluate treatment efficacy, explore secondary outcomes, and identify predictors of success [35]. Ownsworth et al. conducted a RCT of the home‐based programinvolving 50 patients, randomized to immediate intervention (*n* = 27) or waitlist control (*n* = 23). The intervention group demonstrated significantly lower depression on the Montgomery–Asberg Depression Rating Scale and higher existential well‐being, functional well‐being, and global quality of life (Functional Assessment of Cancer Therapy–Brain) compared to controls at post‐assessment. Six‐month follow‐up revealed sustained reductions in depression/stress and improvements in existential well‐being/quality of life, supported by patient‐reported outcomes from validated scales [36].

Primary malignant brain tumor patients impose substantial physical and emotional burdens on family caregivers. Boele et al. investigated an 8‐week nurse‐led online needs support program (SmartCare) compared to enhanced usual care with/without online self‐guided CBT for improving caregiver outcomes. Among 120 caregivers with depression symptom scale scores ≥ 6, intervention groups were merged due to low CBT engagement. Results showed reduced caregiving‐specific distress and a trend toward improved mastery in the SmartCare group, suggesting potential benefits for family quality of life [37].

Caregivers of malignant glioma patients also experience high anxiety rates, yet their distress remains understudied. Forst et al. conducted semi‐structured interviews and thematic content analysis with 21 U.S. caregivers exhibiting clinically significant anxiety (mean age 54.81 years). Six themes emerged, including coping strategies and unmet needs. Caregivers expressed interest in early post‐diagnosis interventions but cited time constraints as barriers, providing critical insights for psychosocial intervention development [38].

CBT targets both emotional and cognitive dimensions of pain. A systematic review incorporating 12 meta‐analyses and 21 studies found that CBT delivered via face‐to‐face or telephone modalities significantly reduced anxiety and depression in cancer populations [39]. ACT has also demonstrated efficacy in improving psychological flexibility and quality of life among cancer survivors, though effects on fatigue and sleep disturbances were less pronounced [40]. Long‐term follow‐up studies indicate that ACT‐induced anxiety reductions persist up to 18 months post‐treatment, with mixed results in direct comparisons to CBT (superiority, equivalence, or modest inferiority reported). Collectively, ACT shows promise as a complementary intervention to enhance psychological resilience and reduce negative affect in cancer survivors [41].

### 3.7 Herbal Medicine

The treatment of CNS tumors, particularly gliomas, remains a medical challenge due to the permeability barriers of the BBB and BTB, which impede chemotherapeutic efficacy and contribute to high mortality rates. Recent investigations into natural compounds and Chinese herbal medicines have yielded novel therapeutic strategies through multitargeted approaches.

Naringenin, a natural flavanone, exhibits anti‐inflammatory, antioxidant, and pleiotropic antitumor properties. It inhibits glioma progression by inducing cell cycle arrest, activating mitochondrial apoptosis pathways, suppressing angiogenesis, and modulating signaling pathways such as Wnt/β‐catenin. Nanocarrier‐based delivery systems enhance their BBB penetration and brain tissue accumulation, while combination therapy with TMZ reverses tumor drug resistance. Emerging evidence indicates naringenin reshapes the tumor microenvironment via STAT3/NF‐κB axis regulation, offering a novel therapeutic strategy for CNS tumors [42].

Celastrol, a pentacyclic triterpenoid extracted from Tripterygium wilfordii, demonstrates broad‐spectrum anticancer activity across multiple malignancies, including brain tumors. Its mechanisms involve inhibiting proliferation/migration, inducing apoptosis, and blocking angiogenesis, with key molecular targets in the PI3K/Akt/mTOR pathway. However, clinical translation is constrained by limited oral bioavailability and a narrow therapeutic window [43].

To circumvent BBB/BTB barriers, Ligusticum chuanxiong essential oil (EO) has been shown to enhance TMZ efficacy in gliomas. Pharmacokinetic analysis and protein expression profiling revealed that EO downregulates P‐glycoprotein (P‐gp) and claudin‐5, promoting TMZ distribution in brain and tumor tissues. Key active components include tetramethylpyrazine and ligustilide [44].

Herbal‐derived bioactive molecules such as catechin, caudatin, and cucurbitacin‐I were identified via bioinformatics screening. Colony formation and CCK‐8 assays showed that these compounds synergistically inhibit GBM T98G cell proliferation when combined with TMZ. Mechanistic studies uncovered activation of the KDELR2‐mediated endoplasmic reticulum stress pathway, validated through qPCR and molecular docking experiments [45].

Traditional herbal combinations also modulate the tumor microenvironment. Astragalus radix (AR) and *Solanum nigrum* (SN) suppress glioma progression by polarizing glioma‐associated microglia/macrophage (GAM). UPLC–QTOF–MS characterization and in vitro/in vivo studies demonstrated ARSN inhibits C6 cell proliferation/migration, promotes apoptosis, and attenuates tumor infiltration. SN exhibits cytotoxic effects, while AR regulates GAM polarization and immune microenvironment remodeling [46].

Additionally, the traditional herbal formula Yunnan Baiyao (YB) exhibits unique mechanisms in glioma resistance. Zhang et al. evaluated YB's antitumor activity and underlying mechanisms using in vitro cell proliferation/colony formation assays with specific inhibitor validation, coupled with in vivo nude mouse and rat glioma models. Results demonstrated that YB induces necroptosis, distinct from conventional apoptotic cell death, in U‐87 MG glioma cells. Further mechanistic investigation revealed that necroptosis induction is contingent upon autophagy, which depends on activation of the AMPK signaling pathway [47].

### 3.8 Endurance and Muscle Training

Miklja et al. investigated the impact of exercise on health‐related quality of life in glioma patients, hypothesizing that individuals with low exercise tolerance would report greater distress in sleep and fatigue domains. Their study included 38 low‐ or high‐grade glioma patients treated at a tertiary medical center. Exercise habits were assessed via telephone interviews, and unpaired *t*‐tests were used for analysis. Results showed that patients with low pre‐illness physical activity levels experienced significantly greater distress in sleep and fatigue, regardless of plasma brain‐derived neurotrophic factor (BDNF) levels. Notably, exercise tolerance did not influence plasma BDNF secretion in adult patients [48].

In a pilot randomized controlled trial, Gehring et al. evaluated the effects of a 6‐month home‐based, remotely coached aerobic exercise intervention in 34 stable glioma patients (WHO grades II–III), comparing it to an active control group. The intervention, consisting of 3 weekly sessions of moderate‐to‐vigorous intensity exercise (20–45 min), led to small‐to‐medium improvements in patient‐reported outcomes, including reduced fatigue, better sleep, improved mood, and mental health‐related quality of life. Cognitive assessments also demonstrated enhancements in attention, information processing speed, and executive function, highlighting the potential of structured exercise to address both cognitive and subjective symptom burdens in this population [49].

Eisenhut et al. conducted a 6‐week study to compare endurance versus strength training interventions in high‐grade glioma (WHO grades III–IV) patients, evaluating physical function and mental health outcomes relative to an active control condition. Findings revealed complex, differential effects of endurance training, strength training, and active control on psychological function, fatigue, sleep, and physical performance 6 weeks post‐neurosurgery/radiochemotherapy. Surprisingly, the active control group demonstrated improvements in multiple metrics and reduced fatigue. Endurance training yielded modest improvements in self‐reported symptoms, while strength training showed no significant changes or adverse trends. However, both exercise groups reported increased fatigue compared to baseline [50].

The BRACE study, a pre‐post phase II intervention, examined the safety, feasibility, and efficacy of an 18‐week individually prescribed exercise program (≥ 150 min/week moderate‐intensity, including resistance training) in 12 adult primary brain cancer survivors. The intervention was deemed safe (no exercise‐related serious adverse events) and feasible (retention 92%, adherence 83%), with significant PRO improvements including quality of life (mean change: 7.9 units, 95% CI: 1.9–13.8), functional well‐being (4.3 units, 95% CI: 1.4–7.2), and reduced depression (−2.0 units, 95% CI: −3.8 to −0.2). Objectively measured outcomes like aerobic fitness (6‐min walk test +56.4 m, 95% CI: 20.4–92.5) and lower‐body strength (+15.2 kg, 95% CI: 9.3–21.1) also improved, underscoring the clinical relevance of tailored exercise in enhancing functional and subjective outcomes [51].

Posterior fossa brain tumors (PFBT) are common pediatric solid tumors. While survival rates have improved with advances in treatment, survivors often experience complications. Although therapeutic exercise has proven beneficial for other pediatric cancer populations, evidence for PFBT survivors remains lacking. The Physical Activity in Childhood Tumor Survivors (PACTS) study enrolled 5–17‐year‐old PFBT survivors at least 12 months post‐surgery and at least 6 months post‐radiochemotherapy. Participants were randomized to receive a 12‐week personalized exercise intervention with family programming. The study assessed cardiorespiratory fitness, goal attainment, and secondary outcomes at multiple time points, aiming to provide critical evidence for pediatric PFBT rehabilitation [52].

Collectively, exercise interventions demonstrate variable efficacy across cancer types and age groups. To address this heterogeneity, future research should focus on optimizing exercise protocols to improve outcomes for cancer patients and survivors.

### 3.9 Dietary Modification and Nutritional Interventions

Cancer development and treatment outcomes are influenced by multiple factors, with dietary regulation emerging as a potentially critical component in CNS tumor management.

Meningiomas, common primary intracranial benign tumors, demonstrate age‐ and gender‐related incidence patterns, with ionizing radiation and genetic syndromes identified as risk factors. Multiple studies show that N‐nitrosocompounds in cured meats may increase meningioma risk, while nitrosation inhibitors in fruits/vegetables (such as high‐protein intake and citrus consumption) exhibit protective effects. However, limited research exists on the Mediterranean diet's role, necessitating multicenter prospective randomized studies to clarify associations [53].

Given the Warburg effect in malignant meningiomas, low‐glucose diets have been explored. Selke et al. investigated low (3 mM), normal (5.5 mM), and high (15 mM) glucose environments plus methylglyoxal on malignant meningioma cell lines (IOMM‐Lee, WHO grade 3) using impedance‐based methods and immunoblotting. Low glucose reduced cell invasion potential, while high glucose impaired barrier function and adhesion, linked to decreased sialic acid content and reduced FAK expression [54].

Gliomas exhibit methionine addiction, monitored via [^11^C]‐methionine PET. Preclinical models show the efficacy of methionine restriction (recombinant methioninase, low‐methionine diet). A 16‐year‐old girl with high‐grade glioma achieved ≥ 60% tumor shrinkage, improved ventricles/sulci, and 19‐month stability without severe toxicity using oral recombinant methioninase combined with a low‐methionine diet alongside radiotherapy/TMZ [55].

Brain tumor patients often require long‐term rehabilitation due to functional decline from lesions/treatment. While protein supplements benefit other cancer populations, evidence for brain tumor patients is scarce. Hee Cho et al. randomized 60 brain tumor patients to 6‐week protein supplementation combined with conventional rehabilitation. The supplement group showed greater improvements in multiple metrics, particularly in malnourished individuals, confirming safety/feasibility [56].

With declining smoking rates, diet and obesity have become critical cancer‐related factors. Obesity correlates with poor outcomes and chronic inflammation. The ketogenic diet (KD), which induces weight loss, anti‐inflammation, and glucose deprivation, shows promise against obesity‐related tumors [57].

Cancer cells rely on aerobic glycolysis (Warburg effect), providing metabolic targets. KD's high‐fat, moderate‐protein, very‐low‐carbohydrate (< 50 g) composition may exploit this dependency through mechanisms including oxidative stress and energy metabolism remodeling. Successful KD implementation requires strict dietary management, with qualified dietitians playing a pivotal role in complication prevention and personalized protocol design [58].

Lysine β‐hydroxybutyrylation (Kbhb), a key post‐translational modification induced by KD, mediates therapeutic effects. Qin et al. identified ALDOB Lys108 as a functional Kbhb site regulated by KD/β‐hydroxybutyrate. Kbhb‐mimetic mutations suppressed cancer cell proliferation, validated through murine hepatic multi‐omic analysis [59].

The Glioblastoma Ketogenic Adjuvant Diet (GLAD), though requiring high adherence, induces meaningful ketonuria and systemic/brain metabolic changes. Importantly, correlations exist between urinary ketones and brain ketone concentrations, with ketonuria providing a more accurate metabolic marker than self‐reported food intake [60].

In a single‐arm phase II trial involving 25 WHO Grade 2–4 astrocytoma patients, GLAD achieved 48% dietary adherence, with 80% of participants attaining moderate ketonuria. Systemic effects included significant reductions in hemoglobin A1c, insulin, and fat mass, alongside increases in lean body mass. Brain magnetic resonance spectroscopy revealed elevated ketone levels (β‐hydroxybutyrate, acetone) in both tumor and contralateral brain regions, reinforcing the diet's ability to modulate central nervous system metabolism [60].

In parallel, a 748‐patient observational study demonstrated histology‐dependent associations between antioxidant intake and survival. Among Grade II/III gliomas, moderate lycopene intake (915.8–2118.3 μg/day) correlated with poorer outcomes (HR = 2.31, 95% CI: 1.12–4.75 for Grade II), whereas high vitamin E (HR = 0.37, 95% CI: 0.17–0.81) and secoisolariciresinol intake predicted improved survival in Grade III cases. Conversely, Grade IV patients exhibited worse prognosis with moderate/high cryptoxanthin (HR = 1.57, 95% CI: 1.20–2.06) or high genistein intake (HR = 1.35, 95% CI: 1.00–1.81), while moderate folate intake (297.4–434.7 μg/day) was associated with better survival in Grade II tumors [61].

## 4 Frontier Technology‐Driven Innovative Paradigm for Integrative Medical Interventions

### 4.1 Practice of Cloud‐Based Multidisciplinary Team (MDT) Ecosystem

Within the innovative paradigm of frontier technology‐driven holistic medical interventions, the exploration of cloud‐based MDT ecosystems holds significant clinical value. MDT management integrates core disciplines, including neurosurgery, medical imaging, pathology, radiotherapy, medical oncology, pharmaceutics, and nuclear medicine, enabling precise treatment planning, improving patient adherence, prolonging survival, enhancing team communication, mitigating medical risks, and fostering interdisciplinary research [62, 63].

For glioma patients with suspected, recurrent, or trial‐eligible tumors, MDT models convene relevant specialists at designated intervals to comprehensively discuss the entire care continuum. Teams composed of a chief specialist, an expert panel, and a coordinator (≥ 5 members) formulate patient‐centered, dynamically adjusted treatment plans. Organizational structures include integrated wards/treatment centers, MDT tumor board meetings, MDT outpatient clinics, and national network platforms, supported by rigorous quality assurance measures. This comprehensive MDT framework ensures optimized care for glioma patients [64].

Karbe et al. confirmed the critical role of surgery in the multidisciplinary treatment of pediatric optic chiasm‐hypothalamic gliomas. Biopsy, hydrocephalus management, exophytic tumor (MRI T2 hyperintensity) resection, and cyst fenestration are key interventions, often performed via minimally invasive techniques like neuronavigated neuroendoscopy. Tumors are classified as endophytic, exophytic, or cystic; the latter two may be surgically amenable under specific conditions. Hydrocephalus can be addressed via third ventriculostomy, stent ETV, or VP shunting, with experienced pediatric neuro–oncology centers incorporating these strategies into systematic multidisciplinary decision‐making systems [65].

Postoperative pituitary adenoma assessment, traditionally conducted in clinical settings, is being transformed by telemedicine. Twenty‐six publications report on mobile applications monitoring parameters for three common complications (adrenal insufficiency, water balance disorders, and visual changes). These apps employ tailored designs and cutting‐edge technologies, leveraging smartphone capabilities to enable remote monitoring. The integration of multidisciplinary technological advancements holds promise for developing customized postoperative pituitary adenoma telemonitoring protocols [66].

Collectively, MDT models demonstrate robust clinical advantages across disease spectrums, while telemedicine innovations enhance postoperative care. Their complementary synergy will drive transformative progress in healthcare delivery and patient outcomes.

### 4.2 Construction of AI‐Driven Intelligent Diagnostic‐Therapeutic Closed‐Loop Systems

**FIGURE 2:**
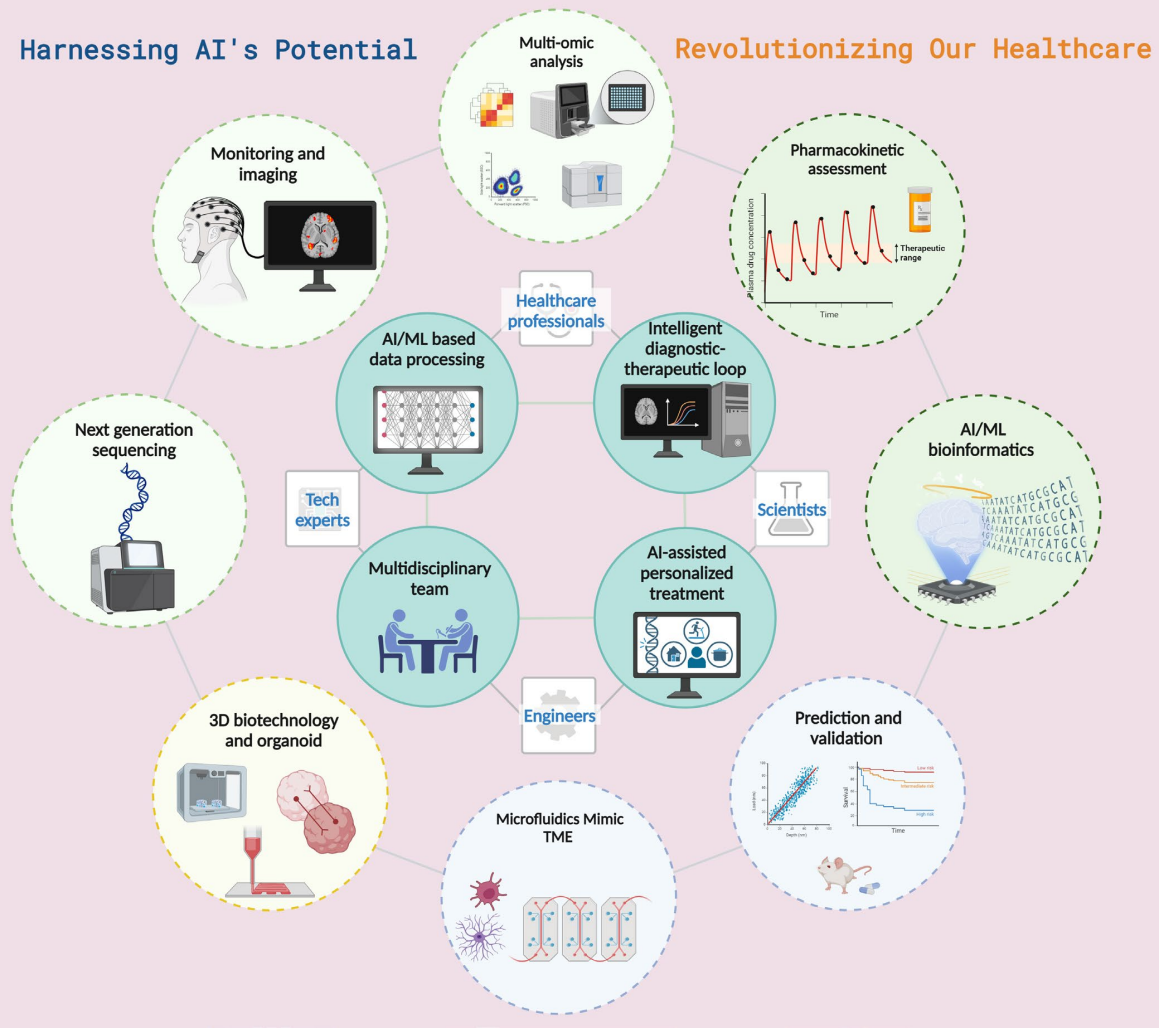
AI‐driven closed‐loop integrated medical intervention system. AI‐driven closed‐loop systems integrate multimodal data to optimize CNS tumor management. Machine learning algorithms analyze imaging, genomics, and real‐time monitoring data to generate adaptive treatment strategies, enabling precision interventions and functional outcome prediction.

In the landscape of frontier technology‐integrated medical interventions, AI‐driven intelligent diagnostic‐therapeutic closed‐loop systems offer transformative potential for optimizing central nervous system CNS tumor management (Figure 2). This potential is realized through the convergence of multiple data‐driven and imaging‐based machine learning approaches.

Real‐world data (RWD) integrated with machine learning has emerged as a cornerstone for patient stratification and prognosis prediction in CNS tumors. For example, leveraging the SEER (Surveillance, Epidemiology, and End Results) database, Yang et al. [67] analyzed 1082 PCNSL cases (1973–2015) using SEER data, integrating clinical features (age, ECOG status, LDH, and CSF protein) to build a nomogram model for cancer‐specific survival. The model showed strong predictive power (C‐index: 0.74 training, 0.72 validation), stratifying patients into low (≤ 40), medium (41–60), and high (≥ 61) risk groups with 3‐year cancer‐specific survival rates of 78.3%, 52.1%, and 28.5%, respectively. This RWD‐driven framework templates multidimensional data (radiomics, genomics, and imaging biomarkers) integration for personalized risk assessment.

Complementing data‐driven strategies, imaging‐based machine learning provides non‐invasive tumor classification solutions. Cao et al. [68] developed a random forest model using 161 patients' MRI data to distinguish low‐grade glioma from GBM. Extracting 78 radiomic features (texture, shape, and intensity) from manually annotated regions of interest, the model achieved 91.3% test accuracy. SHAP analysis identified tumor enhancement, peritumoral edema heterogeneity, and age as key discriminators, with 94.7% accuracy in predicting IDH wild‐type GBM. This highlights RWD‐derived radiomic signatures combined with clinical/genomic data (e.g., IDH status), enabling pre‐surgical malignancy assessment and treatment stratification, addressing the need for non‐invasive precision diagnostics in heterogeneous CNS tumors.

When focusing on glioma diagnosis, an ideal testing system requires high diagnostic accuracy by integrating key biomarkers like IDH1 R132, along with DNA sequence alterations and copy number variations. It must balance sensitivity and specificity to prevent overtreatment while predicting individual glioma susceptibility through comprehensive genetic analysis [69].

For intramedullary gliomas, accurately determining tumor grading and molecular marker status (such as α‐thalassemia/mental retardation syndrome X‐linked and tumor protein P53) is essential for treatment planning and prognosis. However, invasive biopsy methods carry significant risks, and noninvasive pathological typing remains challenging. To overcome this, a Tsinghua University research team combined preoperative sagittal (SAG) and transverse (TRA) T2‐weighted MRI scans with clinical data from 461 patients. Using a transformer‐based deep learning model for automated lesion segmentation and radiomic feature extraction, they compared results with other leading algorithms. The Swin Transformer excelled in SAG/TRA image analysis, while a multimodal fusion feature‐based neural network achieved optimal accuracy in external validation cohorts for predicting intramedullary glioma grading, α‐thalassemia/mental retardation syndrome X‐linked status, and P53 status. This multimodal AI strategy represents the first noninvasive prediction of intramedullary glioma‐related molecular and pathological information, providing strong support for clinical decision‐making and prognostic assessments [70].

### 4.3 Synergistic Delivery Systems for BBB‐Penetrating Targeted Drugs

**FIGURE 3:**
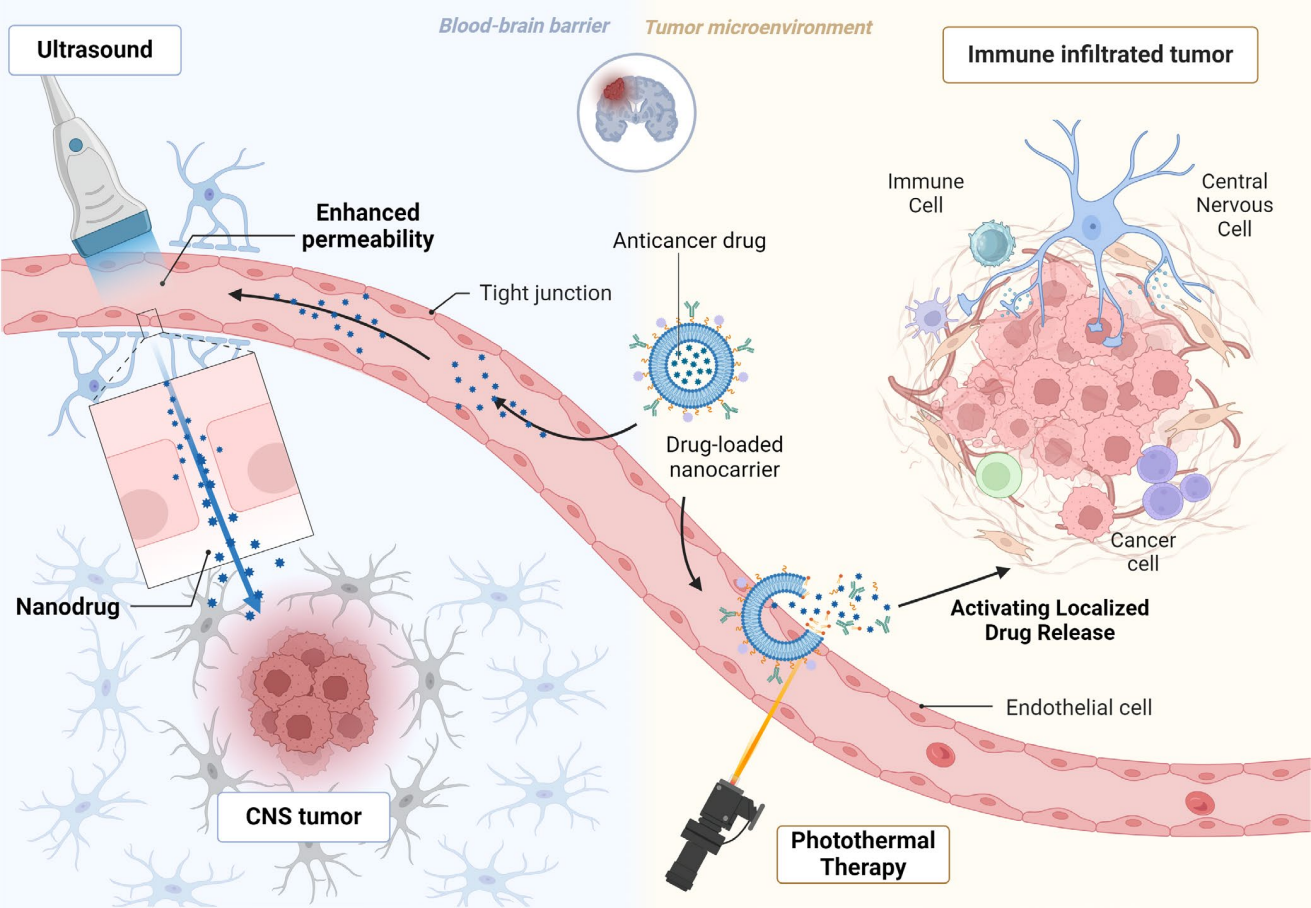
Multi‐technology convergence in BBB penetration and CNS tumor targeting. Synergistic delivery systems combining ultrasound, nanocarriers, phototherapy, and magnetotherapy overcome BBB limitations. FUS enables reversible BBB disruption, while nanocarriers guided by photothermal/magnetic cues achieve spatiotemporally precise drug delivery and immune modulation.

The cardinal challenges in CNS tumor therapy arise from the BBB's physiological restrictions and tumor cell heterogeneity. Conventional drug delivery systems struggle to overcome the BBB's selective permeability, resulting in insufficient brain drug concentrations. Recent advancements in nanocarriers, cell‐penetrating peptides, and virus‐like particles offer innovative solutions for targeted brain delivery, with bispecific antibodies (BsAbs) and nanocarrier‐mediated co‐delivery systems demonstrating particular efficacy (Figure 3).

Receptor tyrosine kinase family member MET acts as a critical signaling hub, with aberrant activation driving glioma malignancy and therapeutic resistance. In GBM, MET hyperactivation remodels the immunosuppressive tumor microenvironment by regulating immune cell dynamics. Transcriptomic analysis reveals strong correlations between MET overexpression, PD‐L1 levels, and phosphorylated STAT4, indicating a MET/STAT4/PD‐L1 axis that reprograms tumor‐associated macrophage (TAM) phenotypes. Functional studies demonstrate that MET inhibitor PLB‐1001 dose‐dependently downregulates PD‐L1 and phosphorylated STAT4 while reducing TAM infiltration, providing rationale for combining MET targeting with immune checkpoint blockade [71].

Notably, significant intertumoral heterogeneity exists in aberrant MET pathway activation. Huang et al. performed molecular profiling of a 485‐glioma cohort using high‐sensitivity RT‐PCR, identifying PTPRZ1–MET (ZM) gene fusions in 14.3% of low‐grade glioma (LGG) and secondary GBM cases, which were undetected in primary GBM. Structural biology studies elucidate that ZM fusion proteins disrupt autoinhibitory conformations, leading to constitutive activation of the MET kinase domain and driving tumor progression to higher grades. This work establishes ZM fusion as a biomarker for LGG progression and underscores the necessity of liquid biopsy‐based early detection to enable precision stratification [72].

Clinically, while MET inhibitor development has advanced, Phase III trial data show variable patient responses, attributed to diverse activation mechanisms (amplification, mutations, and fusions). This necessitates molecularly stratified dosing strategies. Additionally, glioma cells create drug delivery barriers via paracrine HGF/MET‐induced endothelial remodeling, providing a rationale for nanocarrier‐based co‐delivery systems [73].

In immune microenvironment interventions, Yasinjan's team developed EGFRvIII/CD3 BsAb (AG596), a T‐cell engager targeting tumor‐specific EGFRvIII and T‐cell CD3ε. Preclinical studies demonstrate BBB penetration and durable responses in orthotopic GBM models. Phase I trials confirm safety, though the cytokine release syndrome risk requires dosing optimization. Combining BsAbs with MET inhibitors may synergize by activating immunity while blocking compensatory resistance pathways, offering a novel therapeutic paradigm [74].

Epigenetic regulation and novel compounds represent emerging frontiers in oncology, particularly for CNS tumors. Zhou et al. [75] found that HDAC inhibitor suberoylanilide hydroxamic acid enhances GBM radiosensitivity by epigenetically downregulating MMP14, a radioresistance‐associated gene. This finding underscores how emerging epigenetic therapies are reshaping CNS tumor management.

A critical player in this landscape is lysine‐specific demethylase 1, overexpressed in glioblastoma, that drives tumor progression by suppressing tumor suppressor pathways and enhancing DNA repair mechanisms. Preclinical studies demonstrate that lysine‐specific demethylase 1 inhibitors (e.g., ORY‐1001, S2172) reduce glioblastoma stem cell (GSC) viability, impair self‐renewal, and synergize with TMZ by compromising homologous recombination repair. Notably, S2172 exhibits BBB permeability and disrupts MYC/SOX2‐driven stemness, offering a promising strategy for clinical translation [76, 77]. Similarly, KDM1A inhibition via NCD38 sensitizes GSCs to TMZ by inducing DNA damage accumulation, while domatinostat, a class I HDAC inhibitor, selectively targets GSCs without harming differentiated cells [78, 79]. Long non‐coding RNAs (e.g., HOTAIR) further modulate oncogenic pathways in gliomas, highlighting the potential of epigenetic editing tools (e.g., CRISPR‐based enhancer silencing) to suppress EGFR expression and enhance TMZ efficacy [80, 81]. These findings underscore the therapeutic potential of combining epigenetic modifiers with conventional therapies to overcome resistance and improve survival.

Cascio's group identified quinazolin‐4(3H)‐one (QST), a BBB‐penetrating agent cytotoxic to GSC. QST radiosensitizes orthotopic PDX models, improving survival when combined with radiotherapy. While limited by incomplete immune system modeling and species‐specific stability, QST has completed Phase I trials with favorable tolerability, warranting Phase 0 “trigger” trials for adjuvant GBM therapy [82].

Recent breakthroughs in nanoparticle engineering have revolutionized BBB penetration and tumor‐targeted drug delivery. Biomimetic nanoparticles, such as hybrid membrane‐coated systems, exploit natural BBB‐crossing mechanisms to co‐deliver chemotherapeutics while minimizing systemic toxicity [83]. Tetrahedral DNA nanocages functionalized with folate receptors demonstrate enhanced glioma targeting and prolonged circulation, enabling efficient delivery of novel agents like lomitetinib [84]. Additionally, exosome‐based nanoreactors utilize glucose oxidase‐mediated starvation therapy and ROS generation to selectively kill tumor cells, achieving 83% tumor suppression in preclinical models [85]. Magnetic NPs guided by external fields and ultrasound‐responsive carriers further enhance spatiotemporal precision, with dual‐targeting strategies (e.g., anti‐OX40/EGFRvIII bispecific antibodies) improving immune checkpoint inhibitor delivery [86, 87]. These innovations highlight the convergence of nanotechnology, immunology, and imaging to overcome BBB limitations and enable personalized therapy.

In summary, precision MET pathway targeting requires integrating molecular profiling, immune modulation, and delivery innovations with epigenetic regulators and novel compounds. Future research should prioritize liquid biopsy‐based dynamic monitoring, immune‐MET pathway crosstalk, and optimized BBB‐penetrating delivery systems to overcome CNS tumor treatment bottlenecks.

### 4.4 Exploration of Immune Microenvironment Remodeling and Combination Therapies

In CNS tumors, particularly GBM, the complex immune microenvironment remains a critical therapeutic challenge. Recent advances in tumor immunology have shifted focus to technologies for remodeling the immunosuppressive microenvironment.

Metabolic interventions, such as ketogenic diets, reshape the immunosuppressive TME by inducing β‐hydroxybutyrate, which enhances T cell infiltration and suppresses PD‐L1 expression via histone β‐hydroxybutyrylation. RNA–lipid particle aggregates further activate RIG‐I‐mediated innate immunity, converting immunologically “cold” TMEs into immunogenic niches [88, 89].

GBM exhibits extensive myeloid cell infiltration, yet the applicability of peripheral non‐CNS cancer myeloid targets in GBM requires further exploration. Zhong et al. discovered that TREM2 (a key immunosuppressive target in peripheral cancers) exerts unique protective roles in GBM. Single‐cell and spatial sequencing revealed downregulated TREM2 in GBM‐infiltrating myeloid cells, inversely correlating with immunosuppressive myeloid expansion and T‐cell exhaustion. CNS‐enriched sphingolipids were shown to bind TREM2, triggering effective antitumor responses. Clinical data indicated that high TREM2 expression in myeloid cells correlates with improved GBM survival, and TREM2 overexpression not only delays GBM progression but also synergizes with anti‐PD‐1 therapy, underscoring the importance of organ‐specific myeloid remodeling [90].

For CNS solid tumors like GBM, sustaining CAR‐T cell functionality for durable antitumor immunity is critical. Agliardi et al. reported that neither intratumoral IL‐12:Fc injection nor systemic CAR‐T cell infusion alone eradicated advanced tumors in orthotopic glioma models, but their combination yielded significant benefits. The combination reduced CAR‐T cell inhibitory receptor expression, reset endogenous T‐cell balance, promoted effector memory T‐cell accumulation, and remodeled the bone marrow compartment. Similarly, dual‐targeted CAR‐T cells (e.g., IL‐13Rα2/TGF‐β CAR‐T) resist TME‐derived immunosuppression, achieving 60% long‐term survival in GBM‐bearing mice [91]. Notably, single‐dose local injection maintained local efficacy while minimizing systemic toxicity, offering a novel strategy to overcome CAR‐T therapy barriers in GBM [92].

GBM's high invasiveness and viral non‐permissiveness often attenuate the efficacy of oncolytic virus (OV) therapies. Xiao et al. identified CDK4/6 inhibitors as potent chemical enhancers of Zika virus in GBM using a two‐step drug screen of 1416 FDA‐approved compounds. Mechanistically, CDK4/6 inhibition promotes MAVS autophagic degradation, weakening antiviral responses and enhancing OV efficacy. In GBM xenograft models, combination treatment significantly suppressed tumor growth, prolonged survival, and induced immunogenic cell death, providing a novel therapeutic strategy [93].

Nassiri's team conducted a multicenter Phase 1/2 study evaluating the safety and efficacy of OV DNX‐2401 combined with anti‐PD‐1 antibody pembrolizumab in recurrent GBM. Among 49 enrolled patients, treatment was well‐tolerated but failed to meet primary efficacy endpoints. The objective response rate was 10.4%, the 12‐month survival was 52.7%, and the median OS was 12.5 months. Although long‐term survivors were observed, response and resistance mechanisms warrant further investigation. The combination demonstrated safety and survival benefits [94].

In CNS tumors, mutant IDH1 defines distinct glioma molecular subtypes, with IDH1 (R132H) mutations being particularly prevalent. Platten et al. reported results from the NOA16 Phase I trial, a multicenter, open‐label study evaluating an IDH1‐vac peptide vaccine in 33 newly diagnosed Grade 3/4 IDH1 (R132H) astrocytoma patients. The vaccine met primary safety endpoints, with most patients developing immune responses and favorable 3‐year PFS/OS. Immune responses correlated with intratumoral neoantigen presentation, and pseudoprogression occurred frequently [95].

Malignant gliomas exhibit immune evasion and CD47 overexpression, while RNA m5C modification plays critical roles in tumorigenesis. A recent study showed that NSUN5 (a 28S rRNA methyltransferase) enhances tumor‐associated macrophage (TAM) phagocytosis by downregulating β‐catenin in a methyltransferase activity‐dependent manner. NSUN5 interacts with CTNNB1 caRNA, deposits m5C modifications, and promotes its degradation via TET2 oxidation and RBFOX2 recognition. NSUN5 expression is epigenetically suppressed by DNA methylation and inversely correlates with IDH1‐R132H mutations. Blockade experiments revealed that combining CD47 inhibitor RRx‐001 with IDH1‐R132H inhibitor ivosidenib synergistically enhances TAM phagocytosis and glioma clearance, offering a novel combinatorial strategy [96].

### 4.5 Precision Medicine Innovations Guided by Multimodal Imaging and Molecular Subtyping Synergy

The rapid evolution of imaging technologies, coupled with deep integration of artificial intelligence, is driving transformative shifts toward precision and personalized care in CNS tumor management.

Radiomics offers a novel paradigm for glioma precision medicine through high‐throughput extraction of radiomic features from medical images. Its unique advantages include risk stratification, preoperative identification of high‐risk recurrence zones, differentiation of true progression from pseudoprogression, and noninvasive molecular characterization. However, clinical translation faces challenges in reproducibility and generalization, necessitating standardized workflows and multicenter validation [97].

The synergistic application of multimodal imaging techniques (diffusion tensor imaging, magnetic resonance spectroscopic imaging, diffusion‐weighted imaging/perfusion‐weighted imaging, quantitative magnetic resonance imaging) offers a more comprehensive perspective for characterizing tumor features. Specifically, diffusion tensor imaging can assist in the preservation of organs at risk, magnetic resonance spectroscopic imaging enhances the accuracy of target delineation through metabolite analysis, and diffusion‐weighted imaging/perfusion‐weighted imaging, along with quantitative magnetic resonance imaging, provide hemodynamic parameter support for the optimization of radiotherapy plans [98].

Keil et al. analyzed glioma and normal tissues using DCE–MRI, revealing significant correlations between kinetic parameters and microvascular features (VEGF‐A expression, vascular area ratio, and mean vessel size). Statistically significant differences in Ktrans and ve values were observed between prognostic groups, demonstrating DCE‐MRI's potential to characterize tumor microvasculature and BBB abnormalities in infiltrative zones [99].

Based on metabolic reprogramming theory, a surface‐enhanced Raman scattering (SERS) diagnostic system enables noninvasive assessment of CNS tumor infiltration margins by quantifying extracellular acidification. The water droplet array‐pH strategy generates rapid tissue pH maps using ultrapure water, overcoming limitations of traditional pH meters. Integration with IR7p nanostar substrates balances cost‐effectiveness and detection accuracy [100].

The integration of transfer learning has significantly improved imaging processing efficiency: pre‐trained models rapidly generate pharmacokinetic parameter maps, while convolutional neural networks enhance image denoising capabilities. This synergy provides more reliable radiological support for prognosis prediction and treatment planning [101].

Addressing the heterogeneity of central neurocytoma (CN), Xie et al. developed an individualized radiotherapy decision model using postoperative contrast‐enhanced MRI and DTI. The study showed that gross total resection significantly improves patient outcomes, while radiotherapy provides greater survival benefits for subtotal resection patients (especially in atypical CN). The model provides data support for treatment optimization by quantitatively assessing residual tumor volume and white matter tract injury risk [102].

For GBM molecular heterogeneity, Luan et al. integrated TCGA genomic data with TCIA radiomic features to construct the first immune‐related lncRNA–radiomic prognostic model. The model effectively differentiates patient prognostic groups using 4 lncRNA signatures and 2 radiomic features, revealing the critical role of the MET/STAT4/PD‐L1 axis in immune microenvironment regulation. This innovation provides a novel tool for predicting immune therapy responses in GBM [103].

Current advancements in imaging technologies increasingly emphasize multitechnology convergence, with notable examples including the integration of SERS navigation systems and multimodal imaging for intraoperative real‐time tumor margin identification, as well as the synergy between transfer learning and radiomics to enhance model generalization capabilities. Despite these progresses, critical challenges persist in areas such as data standardization and cross‐modal feature fusion, which must be addressed to unlock the full translational potential. Future research should prioritize three key directions: (1) the development of liquid biopsy‐based multi‐omics‐imaging models for dynamic tumor monitoring; (2) the exploration of AI‐driven fully automated optimization algorithms in radiotherapy planning; and (3) the construction of open‐access imaging databases to facilitate multicenter collaborative studies. Collectively, these technological breakthroughs will establish a robust technical foundation for precision diagnostics and therapeutics in CNS oncology, ultimately improving patient outcomes through integrated, data‐driven approaches.

### 4.6 3D Biotechnology and Organoid Models Revolutionizing CNS Oncology Drug Development

CNS tumor research is undergoing a paradigmatic shift from traditional 2D models to advanced 3D bioengineering technologies. This transformation addresses the limitations of conventional systems in recapitulating tumor microenvironment complexity, with emerging 3D culture systems, organoids, and microfluidic chips offering innovative tools to dissect tumor heterogeneity and drug response mechanisms.

Compared to 2D cultures, 3D technologies (e.g., spheroids, organoids) provide more physiologically relevant TME modeling. GBM cells harboring IDH1‐R132H mutations exhibit distinct malignant phenotypes in 3D systems compared to 2D cultures, highlighting TME‐mediated regulation of gene expression and drug resistance. Patient‐derived tumor spheres, neoCOR, and organoid models have enabled drug response testing and therapeutic target screening, though their utility is constrained by the absence of functional vasculature. Notably, brain cancer‐on‐a‐chip platforms overcome this limitation through high‐throughput screening, dynamically monitoring drug penetration efficiency and tumor‐cell interactions [104]. Despite higher costs, 3D cultures provide critical foundational modeling for personalized medicine.

Human organoid technology has opened new frontiers in pediatric brain tumor research (e.g., medulloblastoma, high‐grade glioma). Chiara Lago's team established a hiPSC‐derived brain organoid system generating cerebellar/forebrain organoids over 60–65 days of differentiation, incorporating genome editing to create cancer models. This system recapitulates TME interactions and evaluates treatment efficacy via orthotopic transplantation, serving as a standardized tool for targeted drug development [105].

However, brain tumor organoids (BTOs) face challenges including immature structures, lack of native extracellular matrix, and vascular/immune components. Integration of automated culture systems, multi‐omics quality control standards, and organoid intelligence technologies may enable standardized BTOs for clinical decision support [106].

Microfluidic devices (e.g., tumor‐on‐a‐chip, vascularized tumor‐on‐a‐chip) address traditional model limitations by dynamically mimicking TME parameters. While 2D cultures offer low‐cost simplicity but fail to capture heterogeneity, and 3D cultures improve TME representation but suffer from poor reproducibility, tumor‐on‐a‐chip's real‐time sensor technology monitors tumor growth and drug metabolism, while vascularized tumor‐on‐a‐chip systems reveal metastasis mechanisms through microvascular network modeling [107].

A novel microfluidic system capable of batch‐producing uniform‐sized spheres recently achieved the first microfluidic integration of triple‐culture BBB spheroids. Studies showed compound transport efficiency (e.g., mannitol) correlates strongly with sphere size, offering critical insights for optimizing BBB properties in brain cancer drug development [108].

Xie et al.'s nab‐paclitaxel‐loaded macrophage complex paired with GBM‐on‐a‐chip demonstrates technology convergence potential. This model recapitulates BBB–GBM interactions, showing macrophage‐mediated delivery significantly enhances nab‐paclitaxel efficacy against GBM while polarizing macrophages toward pro‐inflammatory M1 phenotypes, which represents a novel strategy for immuno‐oncology [109].

Future research should prioritize three key directions: (1) developing multi‐scale organoid models incorporating vasculature and immune components; (2) creating microfluidic‐based high‐throughput drug sensitivity testing platforms; and (3) establishing organoid intelligence databases to enable personalized treatment design. Collectively, these technological breakthroughs will propel CNS tumor research from mechanistic discovery to precision medicine implementation.

### 4.7 Sequencing Technology‐Driven Reconstruction of Targeted Diagnosis and Treatment Systems

Breakthroughs in next‐generation sequencing (NGS) technologies, including whole exome sequencing, whole genome sequencing, and RNA sequencing, have established a molecular framework for precision medicine by delineating tumor genomic landscapes. These advances elucidate critical oncogenic mechanisms, exemplified by KRAS co‐mutation patterns in colorectal cancer that drive resistance to EGFR inhibitors. Concurrently, studies on mammalian bombesin (Bn)‐related peptides and their receptors in CNS tumors are uncovering unique molecular regulatory networks, presenting novel therapeutic opportunities when integrated with GBM niche dynamics. However, clinical translation faces dual challenges rooted in tumor heterogeneity. First, disparities in healthcare systems limit universal access to genomic sequencing—the current diagnostic gold standard. Second, conventional randomized clinical trial designs prove inadequate for addressing tumor heterogeneity. To mitigate these barriers, emerging strategies emphasize single‐arm Phase II trials and cross‐tumor drug development frameworks to accelerate therapeutic approval. Future directions advocate for multidimensional approaches combining spatial biology, organoid modeling, and multi‐omics integration to construct heterogeneity maps, thereby enhancing the clinical actionability of genomic data [110]. This paradigm shift underscores the imperative to align technological innovation with adaptive clinical trial designs in the era of precision oncology.

Integrating multi‐omics datasets (genome, transcriptome, epigenome, proteome, and metabolome) has emerged as a powerful strategy to dissect subtype‐specific responses to therapeutic interventions. For instance, a recent study by Lv et al. [111] characterized multifocal GBM using whole‐genome sequencing, bisulfite sequencing, and RNA sequencing, identifying distinct molecular subtypes with heterogeneous alterations in TERT promoter, PTEN, and EGFR mutations, differential methylation patterns (chromosomes 16, 17, and 22), and Hippo/YAP1 signaling dysregulation. Transcriptomic profiling further highlighted subtype‐specific enrichment of extracellular matrix components, immune response genes (LIF, CCL2), and neuron‐interaction pathways, underscoring the TME's role in molecular divergence. In a complementary analysis, Chen et al. [112] classified GBM into disulfidptosis‐related subtypes via genomic and single‐cell sequencing, revealing that Subtype A (high disulfidptosis gene expression) correlated with poor prognosis and CD4^+^ T cells infiltration. Mechanistically, SPAG4‐mediated CD47 upregulation suppressed macrophage phagocytosis, linking cell death pathways to immune evasion. Wang et al. [113] further expanded this framework by integrating genomic, phosphoproteomic, and metabolomic data from 99 treatment‐naïve GBM cases, identifying subtype‐specific signatures in Ras signaling (PTPN11/PLCγ1), metabolic reprogramming (e.g., IDH‐mutant 2‐hydroxyglutarate accumulation), and immune phenotypes (H2B acetylation‐driven subtypes). Notably, glycolysis‐dependent subtypes exhibited enhanced sensitivity to pathway‐targeted therapies, directly connecting molecular heterogeneity to clinical outcomes.

The spatiotemporal heterogeneity of the GBM TME, driven by bidirectional crosstalk between GSCs and stromal components, complicates single‐target therapies. Advanced spatiotemporal multi‐omics technologies now provide unprecedented resolution to dissect niche dynamics, while vascularized organoid models and microfluidic platforms enable biomimetic therapeutic development [2, 114].

Bn‐related peptides and their receptors (BnR) exhibit unique expression patterns and functional characteristics in central nervous system tumors. In gliomas, the abnormal expression of the BnR family is associated with tumor grading and patient prognosis. Antagonists of BnR can provide new therapeutic strategies by inhibiting tumor growth. In neuroblastomas, the Bn/BnR signaling pathway regulates the proliferation and metastasis of tumor cells. The tumor‐suppressive effect of GRPR antagonists and the growth‐promoting effect of GRP endow them with the dual potential of being both diagnostic markers and therapeutic targets. In medulloblastomas, the synergistic expression of GRPR and NMBR may serve as an enhancing target for EGFR cascade reaction inhibitors, but its clinical value still requires further verification [115]. Translational advances include BnR‐targeted imaging probes (68Ga‐IRDye800CW‐BBN, IRDye800CW‐BBN), which achieve superior GBM localization. These probes facilitate preoperative PET/NIRF dual‐modal imaging and intraoperative navigation, enabling 83% gross total resection rates and prolonged survival in clinical cohorts [115].

Collectively, the convergence of NGS with multi‐omics integration is transforming oncology research from single‐molecule studies to systems biology. This paradigm elucidates subtype‐specific vulnerabilities (e.g., ECM remodeling, immune evasion) and resistance mechanisms (e.g., metabolic rewiring), while synergizing Bn/BnR pathway insights, niche modeling, and probe‐based validation to advance personalized CNS tumor management.

## 5 Future Directions of Integrative Medicine Amid Technological Convergence and Ethical Challenges

### 5.1 Evidentiary Challenges in Non‐Pharmacological Therapies

Current integrative medical interventions, particularly non‐pharmacological therapies, face significant evidence gaps in evidence‐based medicine. For example, Traditional Chinese Medicine mind–body therapies for neuropathic pain remain controversial in terms of efficacy, safety, and mechanism. Existing studies on aromatherapy massage and mind‐regulation acupuncture involve fewer than 100 patients per study, with a high risk of reporting bias due to missing registry/protocol information and substantial heterogeneity (e.g., intervention variability, self‐reporting bias), severely limiting generalizability [116]. Only three non‐pharmacological intervention studies on fatigue in primary brain tumor patients meet the criteria, with extremely low evidence quality. Multi‐center, large‐sample RCTs are urgently needed for verification [117].

To address this challenge, a standardized evaluation system must be established to report critical parameters, including treatment efficacy, safety, and intervention frequency/duration for integrative medical modalities. This should involve strengthening clinical trial registration systems to standardize processes such as randomization and allocation concealment, while integrating patient‐reported outcome tools to quantify quality of life and fatigue improvement. Specifically, the Functional Assessment of Cancer Therapy–Brain scale can be used to evaluate brain tumor functional status, with reference to the successful model of tai chi interventions in breast cancer patients [118] to develop CNS tumor‐specific efficacy criteria.

### 5.2 Technical Integration Challenges in Precision Treatment

Precision interventions based on molecular characterization have emerged as a paradigm shift for overcoming CNS tumor heterogeneity. For example, IDH‐mutant glioma patients treated with adjuvant chemoradiotherapy combined with the IDH inhibitor vorasidenib demonstrate significantly prolonged progression‐free survival and improved neurocognitive outcomes [119]. Molecular profiling of pilocytic astrocytoma reveals novel oncogenic drivers such as the GNAI3–BRAF fusion, paving the way for pathway‐specific therapeutic development to advance personalized care [120].

The hallmark of precision intervention lies in the seamless integration of multi‐omics data, where NGS technologies decode key molecular determinants, including IDH1/2 mutations and TERT promoter alterations. These genetic insights are synergistically combined with radiomic signatures (e.g., texture analysis, dynamic contrast‐enhanced parameters) to construct prognostic prediction models, while patient‐derived organoid platforms enable in vitro functional validation of treatment plans. However, this integration faces three critical challenges: first, the heterogeneity of multi‐omics data necessitates standardized interfaces and ontology mapping systems (e.g., using FHIR standards for data interoperability); second, simulating the dynamic evolution of the tumor microenvironment requires combining single‐cell sequencing with in vivo imaging to dissect spatiotemporal interactions between drug delivery and immune responses (e.g., the synergistic mechanism of oncolytic viruses and PD‐1 inhibitors); and third, addressing the low translational efficiency of < 10% demands a tri‐level validation framework integrating organoids, organ‐on‐a‐chip technologies, and patient data. This framework leverages microfluidic systems to simulate BBB permeability, thereby accelerating candidate drug screening.

### 5.3 Synergistic Development of Ethical Governance and Technological Innovation

While technological innovation drives breakthroughs in precision medicine, it concurrently raises profound ethical and societal dilemmas. The exorbitant costs of sequencing technologies and targeted therapies may limit accessibility in low‐income countries, exacerbating healthcare disparities in resource allocation.

The centralized storage of multi‐omics data is confronted with the risk of hacker attacks. Therefore, it is urgent to protect data privacy and combine blockchain technology to ensure the immutability of data. In view of the black‐box decision‐making characteristic of AI diagnosis and treatment systems, it is necessary to develop interpretable models and establish a human–machine collaborative decision‐making mechanism so as to enhance the diagnosis efficiency while safeguarding patients' right to know.

The application of digital therapeutics in CNS tumor care amplifies existing technological divides: AI‐driven early diagnosis models in developed countries demonstrate clinical value, yet model generalization in resource‐limited settings remains constrained by inadequate computing power and inconsistent data annotation quality. This technical inequality risks exacerbating treatment outcome disparities between regions, underscoring the urgent need for standardized international data‐sharing frameworks through global collaboration.

Addressing ethical challenges requires establishing a “3E governance framework” that includes the development of Ethical Guidelines for Precision CNS Oncology to clarify AI decision transparency and data usage boundaries, the implementation of interdisciplinary training programs to enhance clinicians' technical ethics literacy, and the creation of dynamic risk assessment models to pre‐audit treatment plan ethical compliance. Over the next decade, deep integration of nanotechnology, artificial intelligence, and synthetic biology may enable a paradigmatic shift in CNS tumor care from “disease control” to “functional reconstruction”, with patient‐centered full‐lifecycle health management systems achievable through synergistic advancements in technological innovation and ethical governance.

### 5.4 Economic Evaluation and Resource Optimization for Emerging Technology Translation

Emerging technologies such as AI‐driven diagnostics, 3D organoid models, and targeted drug delivery systems necessitate rigorous cost–benefit analyses and pragmatic evaluations to address clinical translation barriers. Lessons from stereotactic laser ablation therapy for brain tumors highlight that optimizing carrier design and enhancing delivery efficiency in targeted drug systems could reduce overall costs by shortening hospitalization and rehabilitation periods, thereby improving cost‐effectiveness ratios [121]. Similarly, intraoperative imaging technologies demonstrate that precision in drug delivery or tumor resection not only improves therapeutic outcomes but also mitigates long‐term costs associated with recurrence and retreatment, provided these technologies achieve high clinical efficacy [122]. For AI diagnostics, establishing multicenter data‐sharing protocols and enhancing algorithmic interpretability—akin to standardizing fluorescence in situ hybridization for 1p/19q co‐deletion detection—are critical to fostering clinical trust and accelerating adoption [123].

Practical challenges in technology dissemination, such as high equipment costs and specialized training requirements, mirror those observed in stereotactic laser ablation and intraoperative imaging. A Markov model‐based analysis of bevacizumab–lomustine combination therapy underscores the importance of aligning cost‐effectiveness ratios with healthcare payment thresholds, suggesting that emerging technologies must demonstrate clear survival benefits or quality‐adjusted life year improvements to justify initial investments [124]. For 3D organoid models, optimizing standardized culture protocols and quality control systems can enhance reproducibility, reducing resource waste and facilitating integration into clinical workflows [122].

Resource allocation strategies must prioritize technologies with proven cost‐effectiveness. The global cost analysis of glioblastoma therapies reveals stark regional disparities in healthcare expenditures, emphasizing the need to tailor adoption strategies to local economic contexts [125]. For instance, ^18^F‐FET PET's superior diagnostic accuracy over MRI in early treatment response assessment justifies its higher upfront costs in settings prioritizing precision medicine [126]. Similarly, centralized production of AI diagnostic platforms or shared organoid biobanks could democratize access while minimizing redundancy. By adopting a tiered resource allocation framework‐prioritizing high‐impact, cost‐efficient technologies–healthcare systems can overcome translational inefficiencies and ensure equitable patient access to innovation.

### 5.5 The Future Landscape of Reinvented Integrative CNS Oncology Care

The future of integrative CNS oncology care will deeply integrate technological innovation with humanistic principles, driving paradigm shifts through multidimensional breakthroughs. Precision interventions based on molecular signatures will leverage multi‐omics data fusion to construct “molecular–imaging–behavioral” digital twin models, enabling real‐time disease monitoring via liquid biopsies, wearable biosensors, and neurointerface technologies. Cloud‐based knowledge graphs will facilitate global real‐time updates and AI‐driven recommendations for multicenter treatment protocols. Innovations in drug delivery systems will focus on biomimetic extracellular vesicle‐mimicking nanocarriers that exploit endogenous BBB penetration mechanisms, combined with photoacoustic/magnetoacoustic dual‐modal guidance for spatiotemporally precise drug release. This approach overcomes chemotherapy's low intracranial bioavailability bottleneck by achieving targeted delivery through BBB‐mimicking transport pathways.

For immune microenvironment remodeling, myeloid cell reprogramming therapies targeting organ‐specific markers like TREM2 and in situ tumor niche vaccine platforms may activate CNS anti‐tumor immunity. Balancing standardization and personalization will require establishing international standards for multi‐modal radiomics and patient‐derived organoid biobanks, with organoid drug sensitivity testing already improving objective response rates in IDH‐mutant glioma patients. Interdisciplinary collaboration will adopt a “clinician–engineer–ethicist” tripartite model [127], coordinated by national research consortia to streamline data sharing and translational research.

As survival rates improve for CNS tumor patients—especially those with low‐grade gliomas and pediatric brain tumors—long‐term follow‐up and quality of life (QoL) analysis have become critical components of integrative care. Existing research highlights the multifaceted challenges faced by survivors, with 30%–50% experiencing severe functional disabilities that impact daily living and work capacity [128]. A preliminary study of 23 glioma survivors (13 employed) revealed that work efficiency was most strongly correlated with self‐reported cognitive function, depression, loneliness, and tumor‐related symptoms, with a regression model explaining 83% of the variance in functional performance. Similarly, non‐work‐related capabilities were linked to cognitive impairment, anxiety, sleep disorders, and symptom burden, underscoring the need for holistic interventions targeting both physical and psychological domains.

Future research should prioritize large‐scale RWD cohorts to identify predictors of late‐onset complications (e.g., cognitive decline, secondary tumors) and QoL decline. Machine learning models integrating patient‐reported outcomes, imaging data, and genomic profiles could dynamically assess risk and tailor interventions—such as cognitive rehabilitation for high‐risk individuals or psychosocial support for those with anxiety. For example, combining the glioma survivor functional model with AI‐driven neuroimaging analysis might identify structural brain changes associated with work disability, enabling proactive interventions. Additionally, leveraging telemedicine and wearable devices for remote QoL monitoring could enhance data collection in real‐world settings, facilitating personalized care that aligns with the integrative medicine goal of “functional reconstruction” rather than mere disease control.

Over the next decade, priority challenges include addressing population aging through integrated patient‐reported outcome tools and digital therapeutics for full‐lifecycle health management [129], while interpretable models enhance AI decision transparency. Patient‐centered care will reduce health disparities via stratified data analytics, cultural sensitivity training, and patient‐family advisory committees [130], ultimately enabling a fundamental shift in CNS oncology from “disease control” to “functional reconstruction.”

## Author Contributions

Junda Lai: writing – original draft. Ketao Liu: writing – review and editing. Yuhua Lin: review and editing. Zhonglin Chen: writing – review and editing. Xianglun Ji: writing – review and editing. Junkai Wen: writing – review and editing, conceptualization, supervision.

## Ethics Statement

The authors have nothing to report.

## Consent

All authors provide consent for the publication of this article.

## Conflicts of Interest

The authors declare no conflicts of interest.

## Data Availability

The data and materials used in this study are available from the corresponding author upon reasonable request.
